# Tryptophan-Starved Human Cells Overexpressing Tryptophanyl-tRNA Synthetase Enhance High-Affinity Tryptophan Uptake via Enzymatic Production of Tryptophanyl-AMP

**DOI:** 10.3390/ijms242015453

**Published:** 2023-10-22

**Authors:** Takumi Yokosawa, Keisuke Wakasugi

**Affiliations:** 1 Komaba Organization for Educational Excellence, The University of Tokyo, 3-8-1 Komaba, Meguro-ku, Tokyo 153-8902, Japan; 2 Department of Life Sciences, Graduate School of Arts and Sciences, The University of Tokyo, 3-8-1 Komaba, Meguro-ku, Tokyo 153-8902, Japan; 3 Department of Biological Sciences, Graduate School of Science, The University of Tokyo, 7-3-1 Hongo, Bunkyo-ku, Tokyo 113-0033, Japan

**Keywords:** aminoacyl-tRNA synthetase, tryptophan, amino acid transport, mutagenesis, tryptophanyl-tRNA synthetase, tryptophan depletion

## Abstract

Our previous study demonstrated that L-tryptophan (Trp)-depleted cells display a marked enhancement in Trp uptake facilitated by extracellular tryptophanyl-tRNA synthetase (TrpRS). Here, we show that Trp uptake into TrpRS-overexpressing cells is also markedly elevated upon Trp starvation. These findings indicate that a Trp-deficient condition is critical for Trp uptake, not only into cells to which TrpRS protein has been added but also into TrpRS-overexpressing cells. We also show that overexpression of TrpRS mutants, which cannot synthesize tryptophanyl-AMP, does not promote Trp uptake, and that inhibition of tryptophanyl-AMP synthesis suppresses this uptake. Overall, these data suggest that tryptophanyl-AMP production by TrpRS is critical for high-affinity Trp uptake.

## 1. Introduction

L-Tryptophan (Trp) metabolizing cells play a crucial role in immune tolerance by eliciting a potent immunosuppressive response. Metabolites associated with Trp catabolism, such as kynurenine (Kyn), in addition to Trp depletion itself, can suppress specific types of immune cells [1,2,3,4,5,6,7,8,9,10,11,12,13,14,15,16,17]. Trp metabolism at the maternal–fetal interface of the placenta acts to guard against rejection of the fetus [1,16]. Some tumors also exploit Trp metabolism-mediated immunosuppression to prevent host immune rejection [5,9,12,13,14,15,17]. For example, indoleamine 2,3-dioxygenase 1 (IDO1), which is the key enzyme that catalyzes the conversion of Trp to Kyn, is overexpressed in some types of cancer and interferon-γ (IFN-γ)-stimulated cells [2,3,4,5,6,7,8,9,10,11,12,14,15,18,19].

The Trp transport system is overexpressed in human IFN-γ-treated or IDO1-expressing cells [18,19,20,21]. Enhanced cellular uptake of Trp results in a loss of extracellular Trp, thereby initiating immune suppression/immune tolerance [1,2,3,4,5,6,7,8,9,10,11,12,13,14,15,16,17]. Specifically, the reduction in the level of extracellular Trp prevents T-cell proliferation, which is the basis for the mechanism of immunosuppressive function [1,2,3,4,5,6,7,8,9,10,11,12,13,14,15,16,17]. Therefore, the Trp uptake mechanism is a potential target as a cancer therapeutic. Previously, we showed that IDO1 and tryptophanyl-tRNA synthetase (TrpRS) are required for elevated Trp uptake into cells [19]. Our study established that human cells treated with IFN-γ showed upregulated levels of IDO1 and TrpRS, which enhanced Trp uptake [19]. Moreover, the level of Trp uptake was decreased by suppression of TrpRS or IDO1 expression using siRNA but increased by overexpression of TrpRS or IDO1 [19].

The expression of human TrpRS is specifically enhanced by IFN-γ [22,23,24,25,26,27]. TrpRS mediates the aminoacylation of Trp onto tRNA^Trp^ [28,29]. This reaction involves the generation of tryptophanyl-AMP from Trp and ATP with concomitant production of inorganic pyrophosphate (PPi) [28]. The activated aminoacyl moiety is then moved from tryptophanyl-AMP to the tRNA^Trp^ to give tryptophanyl-tRNA^Trp^ and AMP [28]. There are two splice variants of human TrpRS: the predominant full-length TrpRS and a minor truncated TrpRS lacking the N-terminal WHEP region [30,31]. In an earlier study, we demonstrated that only the truncated form of TrpRS can act as an angiostatic factor [32]. Thus, TrpRS has additional roles other than tRNA aminoacylation [32,33,34]. Both full-length and truncated TrpRSs are secreted from cells [33,35,36,37].

In a previous study, we showed that incorporation of TrpRS protein into the assay buffer induced HeLa cells to upregulate Trp uptake [19,38]. Moreover, overexpression of a Trp-metabolizing enzyme, either IDO1 or tryptophan 2,3-dioxygenase (TDO2), significantly increased TrpRS-mediated Trp uptake [38]. Overall, the study showed that Trp depletion, in contrast to the production of Kyn, is important for extracellular TrpRS-mediated Trp cellular uptake [38]. However, it remains unclear whether Trp starvation is important for Trp uptake into TrpRS-overexpressing cells. Furthermore, the molecular mechanism of Trp uptake by TrpRS is unknown.

Here, we investigated whether high-affinity Trp uptake into TrpRS-overexpressing cells is affected by the Trp starvation state of the cells. We also examined the importance of tryptophanyl-AMP production by TrpRS in terms of TrpRS-mediated Trp uptake.

## 2. Results

### 2.1. Trp Depletion Enhances Trp uptake into TrpRS-Overexpressing HeLa Cells

**Figure 1:**
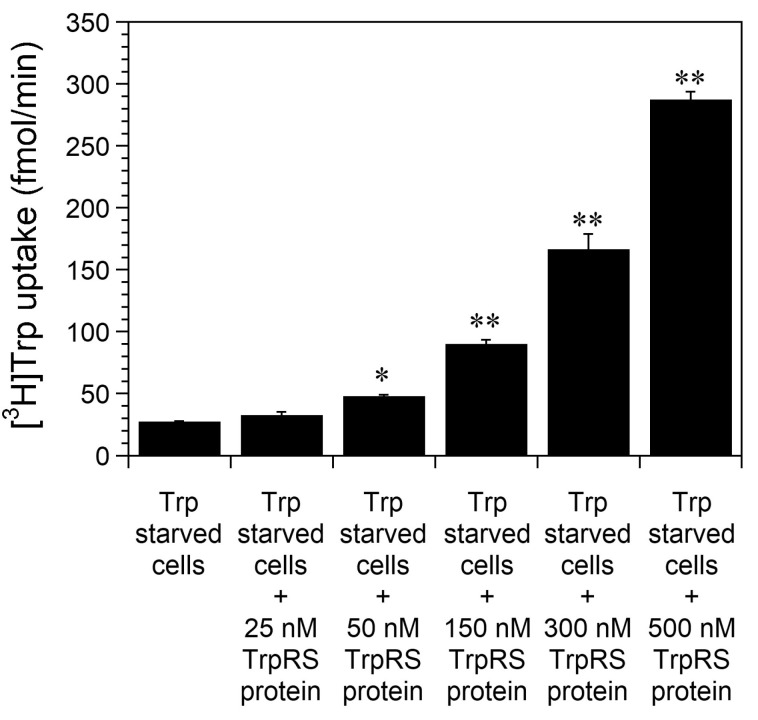
The effect of concentration dependence of TrpRS protein on high-affinity Trp uptake into Trp-starved HeLa cells. HeLa cells were incubated in Trp-free medium for 24 h prior to measuring cellular uptake of [^3^H]Trp in the presence of the indicated amount of human TrpRS protein. All data are expressed as the mean ± standard deviation (SD) of four separate experiments. ** *p* < 0.01, * *p* < 0.05.

Our previous study demonstrated that Trp uptake into cells facilitated by extracellular TrpRS protein is enhanced by Trp depletion via Trp-metabolizing enzymes [38]. In this study, we examined the concentration dependence of exogenous TrpRS protein on Trp uptake into Trp-starved cells. Specifically, HeLa cells were initially incubated in Trp-free cell culture medium for 24 h prior to the addition of human TrpRS protein. The uptake of [^3^H]Trp was then monitored. A marked enhancement in Trp uptake into Trp-starved cells was evident in the presence of 500 nM TrpRS protein in comparison to the control (no TrpRS) (Figure 1). The addition of lower levels of TrpRS (300 nM, 150 nM, 50 nM) resulted in a progressively reduced enhancement in Trp uptake and 25 nM TrpRS protein had almost no effect on Trp uptake (Figure 1). 

**Figure 2:**
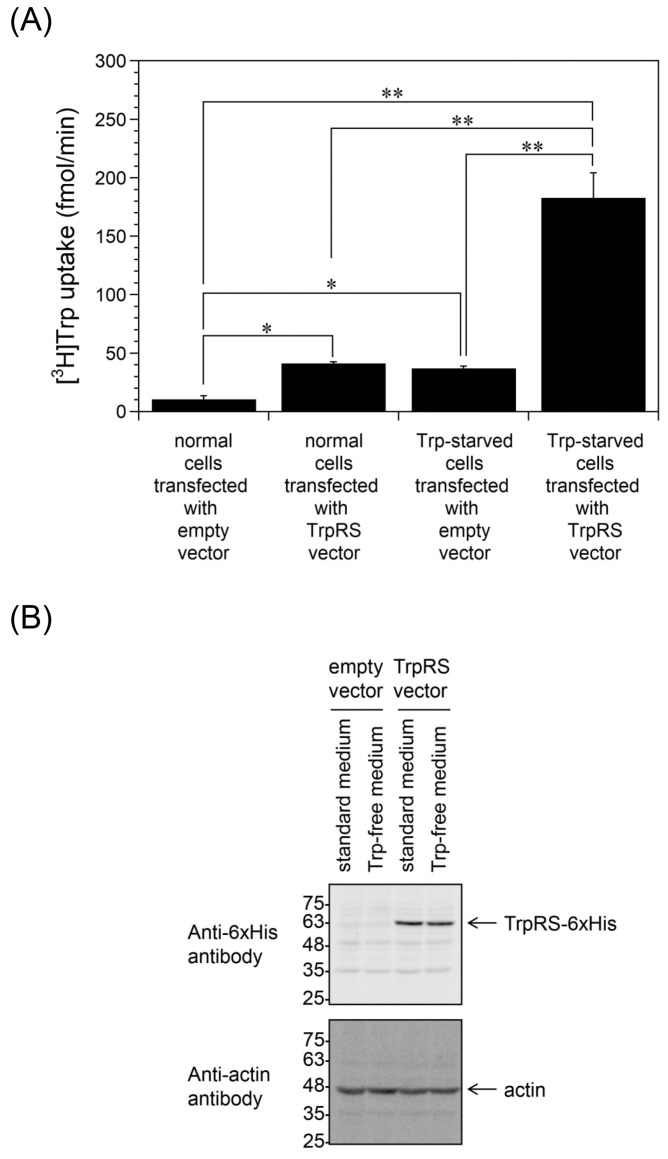
Trp uptake into TrpRS-overexpressing HeLa cells, which had been incubated in either standard or Trp-free medium. (**A**) HeLa cells overexpressing human TrpRS were incubated in standard or Trp-free medium for 24 h prior to measuring Trp uptake. All data are expressed as the mean ± SD of three or four independent experiments. ** *p* < 0.01, * *p* < 0.05. (**B**) Western blot analyses of HeLa cells overexpressing human TrpRS incubated in either standard or Trp-free medium. Migration positions of protein markers (kDa) are indicated on the left-hand side. Actin was used as a loading control.

Our previous work revealed that overexpression of TrpRS promotes Trp uptake into HeLa cells [19]. Here, we examined whether Trp depletion is important for Trp uptake into TrpRS-overexpressing HeLa cells. The HeLa cells were transfected with either an empty vector or human TrpRS expression vector and incubated in standard or Trp-free culture medium for 24 h. Consistent with the previous study, overexpression of TrpRS in HeLa cells cultured in standard medium increased Trp uptake rates compared to the empty vector-transfected cells (Figure 2A). In addition, TrpRS-overexpressing cells incubated in Trp-free medium displayed enhanced levels of Trp uptake compared to TrpRS-overexpressing cells incubated in standard medium or empty vector-transfected cells incubated in Trp-free medium (Figure 2A). Western blotting indicated that the expression level of polyhistidine-tagged human TrpRS (55 kDa) in Trp-starved cells was almost the same as that of normal cells (Figure 2B).

### 2.2. TrpRS Facilitates High-Affinity Trp Uptake by Production of Tryptophanyl-AMP

**Figure 3:**
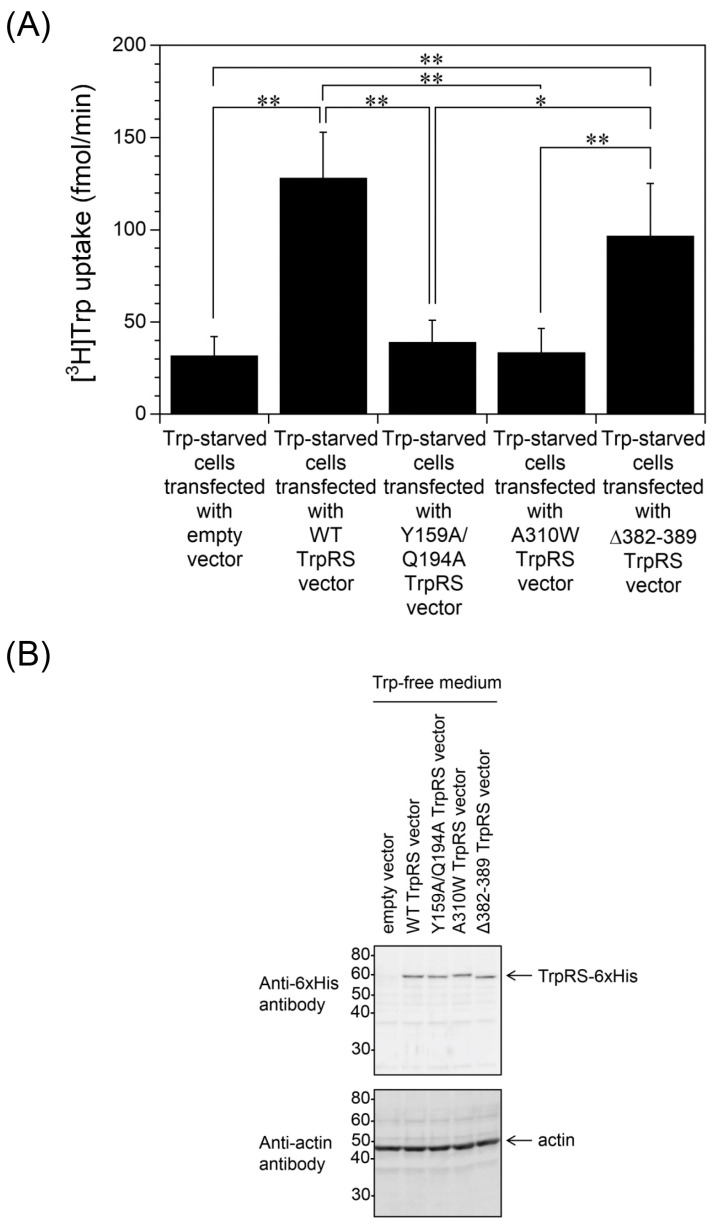
Trp uptake into Trp-starved cells overexpressing human WT TrpRS or TrpRS mutant. (**A**) Trp uptake into cells overexpressing human wild-type (WT) TrpRS or TrpRS mutant, which had been grown in Trp-free medium for 24 h. All data are expressed as the mean ± SD of three or four independent experiments. ** *p* < 0.01, * *p* < 0.05. (**B**) Western blot analyses of HeLa cells overexpressing human WT TrpRS or TrpRS mutant, which had been grown in Trp-free medium for 24 h. Migration position of protein markers (kDa) are indicated on the left-hand side. Actin was used as a loading control.

For human TrpRS, the regions of the protein that bind Trp and ATP, in contrast to the tRNA-binding site, can regulate cellular Trp uptake [19]. Here, we assessed whether the Trp- and the ATP-binding sites of TrpRS are also required for Trp uptake into Trp-starved cells. Specifically, HeLa cells, which had been transfected with an expression vector encoding mutant versions of TrpRS, were incubated in Trp-free medium for 24 h and Trp uptake was measured. Overexpression of Y159A/Q194A double mutant TrpRS, which cannot bind to Trp, or A310W TrpRS, in which the AMP pocket is inaccessible [39], did not increase Trp uptake into Trp-starved cells (Figure 3A). Importantly, these TrpRS mutants are unable to generate tryptophanyl-AMP. Δ382–389 TrpRS is a tRNA binding-deficient mutant [40], meaning that this TrpRS mutant can generate tryptophanyl-AMP but cannot aminoacylate tRNA. Overexpression of the Δ382–389 mutant enhanced Trp uptake into Trp-starved cells to a similar level as wild-type (WT) TrpRS-overexpressing cells (Figure 3A). The presence of polyhistidine-tagged human TrpRSs in HeLa cells was confirmed by Western blot analysis using an anti-polyhistidine antibody (Figure 3B). These results suggest that the Trp- and ATP-binding sites, but not the tRNA-binding ability, of human TrpRS protein are essential for Trp uptake into Trp-starved cells.

**Figure 4:**
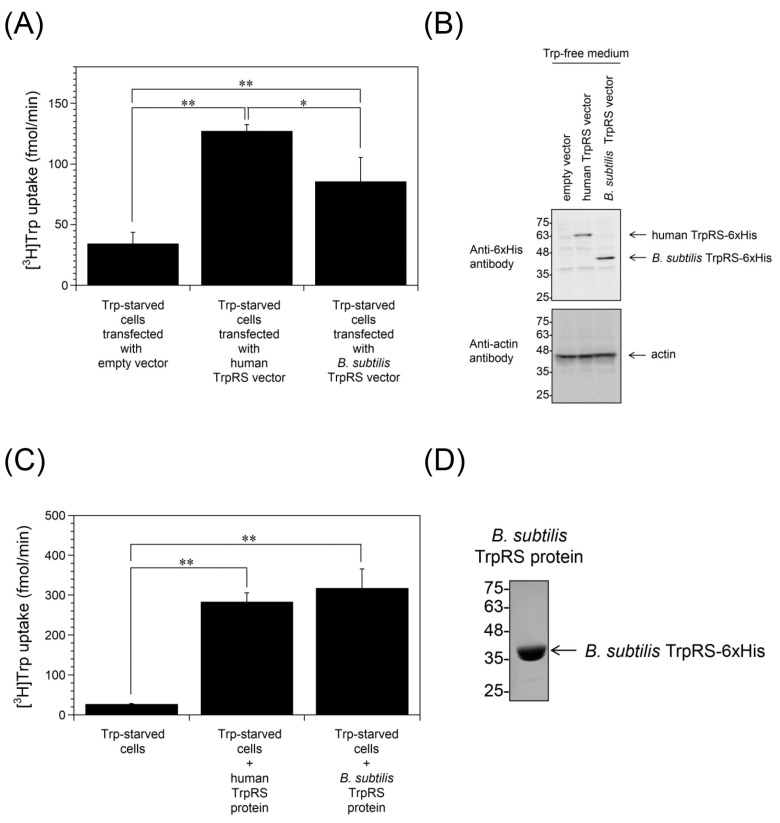
Trp uptake into Trp-starved HeLa cells overexpressing human or *B. subtilis* TrpRS and into Trp-starved HeLa cells in the absence or presence of human or *B. subtilis* TrpRS protein. (**A**) Trp uptake into HeLa cells overexpressing human or *B. subtilis* TrpRS, which had been grown in Trp-free medium for 24 h. All data are expressed as the mean ± SD of three independent experiments. ** *p* < 0.01, * *p* < 0.05. (**B**) Western blot analyses of HeLa cells overexpressing TrpRS, which had been grown in Trp-free medium for 24 h. Migration position of protein markers (kDa) are indicated on the left-hand side. Actin was used as a loading control. (**C**) Trp uptake into Trp-starved HeLa cells in the absence or presence of 500 nM human or *B. subtilis* TrpRS protein. All data are expressed as the mean ± SD of four or five independent experiments. ** *p* < 0.01. (**D**) SDS-PAGE analysis of purified *B. subtilis* TrpRS protein after staining with CBB. Migration positions of protein markers (kDa) are indicated on the left-hand side.

Trp uptake into *Bacillus subtilis* TrpRS-overexpressing cells starved of Trp was also measured. As shown in Figure 4A, high-affinity Trp uptake was enhanced in *B. subtilis* TrpRS-overexpressing Trp-starved cells as observed with human TrpRS-overexpressing cells. Overexpression of polyhistidine-tagged *B. subtilis* TrpRS (40 kDa) in HeLa cells was confirmed by Western blot analysis (Figure 4B). Incorporation of purified *B. subtilis* TrpRS protein into the assay buffer also enhanced Trp uptake into Trp-starved cells (Figure 4C). Recombinant *B. subtilis* TrpRS was generated in *Escherichia coli* and purified to near homogeneity. The protein preparation was assessed by SDS-polyacrylamide gel electrophoresis (SDS-PAGE). A band matching polyhistidine-tagged *B. subtilis* TrpRS was observed after staining with Coomassie Brilliant Blue (CBB) (Figure 4D). Prokaryotic and eukaryotic cytoplasmic TrpRSs are unable to cross-aminoacylate their respective tRNA^Trp^ [41]. Therefore, *B. subtilis* TrpRS can generate tryptophanyl-AMP but not aminoacylate human tRNA^Trp^ in HeLa cells. These observations suggest that TrpRS facilitates Trp uptake into cells in a manner that depends on its ability to produce tryptophanyl-AMP.

**Figure 5:**
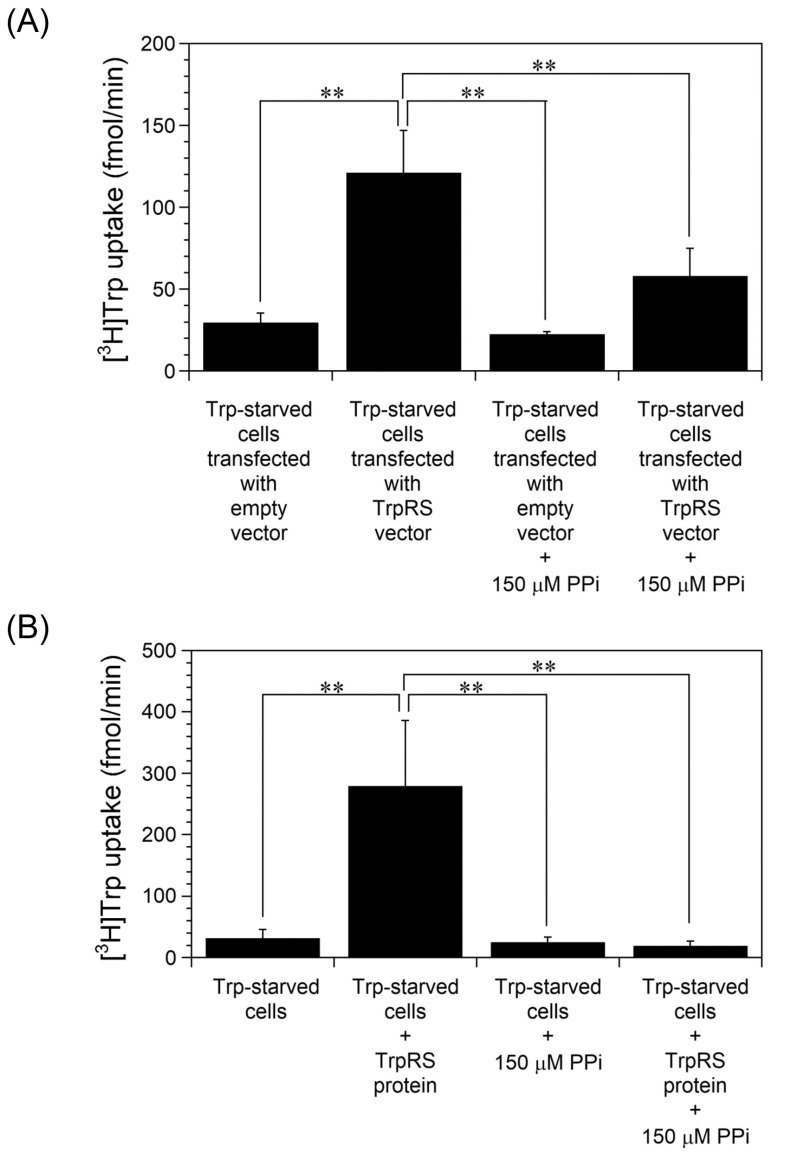
Effect of PPi on high-affinity Trp uptake into Trp-starved HeLa cells. (**A**) Trp uptake into human TrpRS-overexpressing cells, which had been grown in Trp-free medium for 24 h, was investigated in the absence or presence of 150 µM PPi. All data are expressed as the mean ± SD of three independent experiments. ** *p* < 0.01. (**B**) Trp uptake into HeLa cells, which had been grown in Trp-free medium for 24 h, was investigated in the absence or presence of 150 µM PPi and 500 nM human TrpRS protein. All data are expressed as the mean ± SD of three independent experiments. ** *p* < 0.01.

Next, the effect of PPi on Trp uptake into cells was investigated. PPi is a general aminoacyl-tRNA synthetase inhibitor [42]. Aminoacyl-tRNA synthetases are responsible for the transesterification of amino acids to their cognate tRNAs in a two-step reaction [29]. Initially, the amino acid is activated by reaction with ATP to generate aminoacyl-AMP plus PPi. In the second stage, the activated aminoacyl moiety is transferred to the corresponding tRNA with release of AMP. PPi can shift the equilibrium of the first reaction toward degrading aminoacyl-AMP in aminoacyl-tRNA synthetases, and then prevent transfer of the amino acid to tRNA. In order to investigate whether TrpRS-mediated Trp uptake into cells is affected by inhibition of tryptophanyl-AMP synthesis, PPi was added to the uptake assay buffer. As shown in Figure 5A, Trp uptake into Trp-starved human TrpRS-overexpressing cells was significantly inhibited by the addition of PPi. Moreover, extracellular human TrpRS protein-mediated Trp uptake into Trp-starved cells was also inhibited upon addition of PPi into the buffer (Figure 5B).

### 2.3. Inhibition of Trp Uptake into Trp-Depleted TrpRS-Overexpressing HeLa Cells

**Figure 6:**
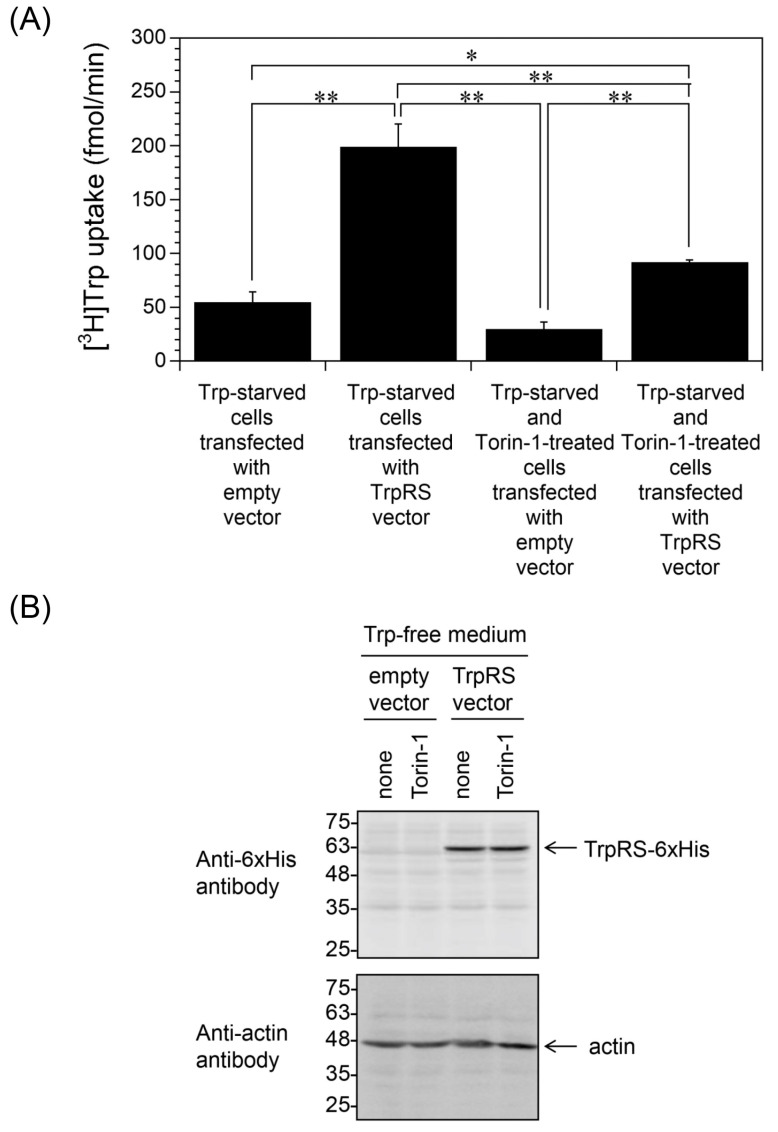
Trp uptake into Trp-starved and Torin-1-treated HeLa cells overexpressing human TrpRS. (**A**) Trp uptake into human TrpRS-overexpressing HeLa cells, which had been grown in Trp-free medium supplemented with 250 nM Torin-1 dissolved in dimethyl sulfoxide (DMSO) or 0.1% DMSO as a control (none) for 24 h. All data are expressed as the mean ± SD of three or four independent experiments. ** *p* < 0.01, * *p* < 0.05. (**B**) Western blot analyses of HeLa cells overexpressing human TrpRS, which had been grown in Trp-free medium in the presence of 250 nM Torin-1 dissolved in DMSO or 0.1% DMSO as a control (none). Migration positions of protein markers (kDa) are indicated on the left-hand side. Actin was used as a loading control.

Next, we investigated the inhibition of Trp uptake. In Trp-depleted cells, ribosomes bypass Trp codons in mRNA and Trp-to-phenylalanine (Phe) codon reassignment occurs [43,44,45]. The resulting aberrant peptides are presented at the cell surface and recognized by T-lymphocytes [43,44,45]. The ribosomal frame-shifting is suppressed by MAPK pathway inhibitors such as Torin-1, a potent inhibitor of a mechanistic target of rapamycin complex 1/2 (mTORC1/2) [44]. Here, we investigated whether Torin-1 affects high-affinity Trp uptake. TrpRS-overexpressing HeLa cells were incubated in Trp-free medium in the absence or presence of Torin-1. As shown in Figure 6A, Torin-1 treatment inhibited Trp uptake into Trp-depleted TrpRS-overexpressing HeLa cells. Western blot analyses established that Torin-1 had no effect on TrpRS expression in the cells (Figure 6B).

## 3. Discussion

In this study, we demonstrate that Trp depletion enhances high-affinity Trp uptake into TrpRS-overexpressing cells. Taking into account our previous experimental results [38], we conclude that Trp-deficient conditions are critical for high-affinity Trp uptake, not only into cells with added TrpRS protein, but also into TrpRS-overexpressing cells. Our findings show that TrpRS mutants deficient in tryptophanyl-AMP production do not enhance high-affinity Trp uptake. Moreover, inhibition of tryptophanyl-AMP synthesis by PPi was found to inhibit Trp uptake. These results demonstrate that TrpRS mediates high-affinity uptake via tryptophanyl-AMP production.

Although tryptophanyl-AMP production was demonstrated to be crucial in TrpRS-mediated cellular Trp uptake, the role of tryptophanyl-AMP in the Trp uptake mechanism remains unclear. Protein aminoacylation, which is a type of posttranslational modification, has been reported as a potential mechanism involving aminoacyl-tRNA synthetases and aminoacyl-AMPs [46]. As well as catalyzing canonical tRNA charging, each aminoacyl-tRNA synthetase senses its cognate amino acid levels via aminoacylation of lysine (Lys) residues on specific substrate proteins via generation of aminoacyl-AMP [46]. For example, TrpRS catalyzes tryptophanylation of thyroid hormone receptor interacting protein 12 (TRIP12), which is an E3 ubiquitin-protein ligase [47]. Tryptophanylation of TRIP12 by TrpRS activates E3 ligase activity of TRIP12, and activated TRIP12 promotes degradation of nuclear factor of activated T-cells 1 (NFATc1), which is a transcription factor that induces expression of programmed death receptor-1 (PD-1), and downregulates PD-1 expression in T-cells [47]. By contrast, Trp starvation may cause a decrease of tryptophanylation of TRIP12 by TrpRS, leading to lower levels of NFATc1 ubiquitination, which increases the amount of NFATc1. Therefore, tryptophanylation by TrpRS and Trp starvation are key to the regulation of TRIP12 activity and NFATc1 expression. Because tryptophanyl-AMP production by TrpRS is important in high-affinity Trp uptake, TrpRS may mediate high-affinity Trp uptake by protein tryptophanylation. For example, extracellular TrpRS may tryptophanylate cell surface molecules and enhance high-affinity Trp uptake into cells. Further work is required to examine the relationship between protein tryptophanylation by TrpRS and the Trp uptake system.

Although it has been shown that Trp uptake is not inhibited by amino acid transport inhibitors [18,19,21], we showed that Torin-1 treatment suppresses high-affinity Trp uptake. Torin-1 inhibits mTORC1/2 and induces autophagy [44]. Because TrpRS expression levels were unaffected by Torin-1 treatment, other factors involved in high-affinity Trp uptake, for example the cell-surface TrpRS-interacting molecules, may be degraded by autophagy. Moreover, Torin-1 is reported to suppress ribosomal frame-shifting in Trp-starved cells [44]. When Trp is depleted by Trp-metabolic activity of IDO1 in cells, ribosomes bypass Trp codons of mRNA and aberrant peptides generated by frame-shifting are presented at the cell surface [43,44]. Therefore, suppression of frame-shifting by Torin-1 might have reduced Trp uptake into Trp-starved cells. In addition to frame-shifting, it is reported that Trp-to-Phe codon reassignment occurs in Trp-starved cells [45]. In Trp-starved cells, Phe may be used instead of Trp for tRNA aminoacylation and protein synthesis by TrpRS [45]. Akin to frame-shifted products, Trp-to-Phe substituted peptides or proteins are presented at the cell surface [45]. Because frame-shifting at Trp codons and Trp-to-Phe substitutions are Trp starvation-specific phenomena, they may be involved in high-affinity Trp uptake. To elucidate the molecular details of high-affinity Trp uptake via TrpRS, the relationship between Trp starvation-specific phenomena and high-affinity Trp uptake should be investigated.

Because Trp uptake is exploited by some cancers to avoid immune rejection by T-cells, inhibition of this process could be the basis for the development of new anti-cancer drugs. Indeed, it has been shown that a cocktail of IDO1 inhibitors and immunotherapeutic drugs, including agents targeting cytotoxic T lymphocyte-associated protein 4 (CTLA4) or PD-1, displays a synergistic effect in combating cancer [48,49]. Elucidation of the mechanism of high-affinity Trp uptake may lead to the generation of novel cancer drugs.

## 4. Materials and Methods

### 4.1. Chemicals

Glycine (Gly), glutamine (Gln), and Trp were obtained from FUJIFILM Wako Pure Chemical Corp. (Osaka, Japan). All other L-amino acids used in this study along with PPi were purchased from Sigma-Aldrich (St. Louis, MO, USA). L-[5-^3^H]Trp ([^3^H]Trp) was obtained from American Radiolabeled Chemicals Inc. (St. Louis, MO, USA). Torin-1 was purchased from ChemScene (Monmouth Junction, NJ, USA).

### 4.2. Cell Culture

HeLa cells (RCB0007) were provided by the RIKEN Cell Bank (Tsukuba, Japan). Cells were cultured in high glucose Dulbecco’s modified Eagle’s medium (DMEM) (Thermo Fisher Scientific, Waltham, MA, USA), 10% (*v*/*v*) fetal bovine serum (FBS) (Biosera, Nuaille, France), and 2 mM Gln. Culturing was performed under standard conditions in 5% CO_2_ at 37 °C.

### 4.3. Western Blot Analyses

Protein samples were analyzed by SDS-PAGE (12% gel) and then electroblotted onto a PVDF membrane (Merck Millipore, Darmstadt, Germany). During blocking, the membrane was soaked in 10 mM Tris-HCl, pH 8.0, 0.1% Tween 20, and 5% skimmed milk (FUJIFILM Wako Pure Chemical Corp.). Membranes were incubated for 1 h in phosphate-buffered saline (PBS, pH 7.4) supplemented with either a mouse monoclonal antibody targeting the polyhistidine tag (Thermo Fisher Scientific), or rabbit anti-β-actin monoclonal antibody (GeneTex Inc., Irvine, CA, USA). The membranes were thoroughly washed in buffer (10 mM Tris-HCl, 0.1% Tween 20, pH 8.0) to remove unbound primary antibody prior to incubation with an HRP-linked secondary antibody (F(ab’)_2_ donkey anti-rabbit IgG or sheep anti-mouse IgG (GE Healthcare Life Sciences, Amersham, UK)) for 1 h. After washing with buffer, the positive bands were detected using ECL™ reagents (GE Healthcare Life Sciences). Chemiluminescent signals were acquired using a LAS-4000 system (GE Healthcare Life Sciences).

### 4.4. Quantification of Trp Uptake

Trp uptake experiments were performed as previously reported [19,38]. In detail, the HeLa cells were extensively washed (3×) in PBS (pH 7.4), and then resuspended in uptake assay buffer (PBS plus 0.3 mM MgCl_2_) to give 1 × 10^6^ cells/mL. [^3^H]Trp (150 nM) was added to a 0.3 mL aliquot of cell suspension plus or minus 500 nM TrpRS protein. Cellular uptake of Trp was then monitored at 25 °C over a series of timepoints (0, 1, 2, 3, and 4 min). Specifically, 50 μL samples were subjected to rapid vacuum filtration through a glass microfiber filter (GE Healthcare Life Sciences) with a particle retention of 1.2 μm. Filters were washed (5×) with 5 mL PBS, air-dried, and analyzed on a scintillation counter (PerkinElmer, Waltham, MA, USA). Trp uptake increased over time in a linear fashion. Uptake was found to be linear, and the initial rate was calculated (fmol min^−1^).

### 4.5. Plasmids

Open reading frames (ORFs) of human TrpRS (amino acids 1–471) and *B. subtilis* TrpRS (amino acids 1–330) were individually engineered into a mammalian expression vector (pcDNA3.1/myc-His(-) B) [19]. The resultant recombinant gene products included a C-terminal polyhistidine (6× His) tag [19]. The ORF of human or *B. subtilis* TrpRS was also subcloned into the prokaryotic expression vector pET-20b (Novagen, Madison, WI, USA), which generated a recombinant product containing a C-terminal polyhistidine (6× His) tag [19,38,50,51,52,53,54]. Mutated versions of human TrpRS were produced using the QuikChange™ kit (Stratagene, La Jolla, CA, USA) to introduce base substitutions at specific sites [19]. Nucleotide sequences of all the constructs were verified prior to further analysis.

### 4.6. Plasmid Transfection Procedure

HeLa cells were seeded at 1.5 × 10^5^ cells/mL in 60 mm dishes (Corning, Corning, NY, USA) 24 h prior to performing the experiment. Polyethylenimine (PEI) “Max” (Polysciences, Warrington, PA, USA)-mediated transfection was carried out using the appropriate constructs. Typically, 5 μg of DNA and 0.02 mL of PEI (1 μg/μL) were mixed in 0.4 mL of a 150 mM NaCl solution. After incubation for 20 min at 25 °C, the mixture was added to the cells. The cells were incubated for 24 h post transfection.

### 4.7. Trp Starvation of HeLa Cells

Trp sarvation of HeLa cells were induced as previously reported [38]. In detail, HeLa cells were seeded in 60 mm dishes (Corning) and incubated for 24 h. Cells were washed (3×) in PBS and incubated for 24 h in amino acid-free DMEM medium (high glucose) (#048-33575; FUJIFILM Wako Pure Chemical Corp.) supplemented with 10% (*v*/*v*) US dialyzed FBS (GE Healthcare Life Sciences), 2 mM Gln, and amino acids (0.4 mM arginine, 0.2 mM cysteine, 0.4 mM glycine, 0.2 mM histidine, 0.8 mM isoleucine, 0.8 mM leucine, 0.8 mM lysine, 0.2 mM methionine, 0.4 mM phenylalanine, 0.4 mM serine, 0.8 mM threonine, 0.4 mM tyrosine, 0.8 mM valine). Note, with the exception of Trp, the concentration of each of the amino acids corresponds to that of standard DMEM (#10313-021; ThermoFisher Scientific).

### 4.8. Preparation of TrpRS Proteins from E. coli

pET-20b encoding either human TrpRS or *B. subtilis* TrpRS was used to transform *E. coli* BL21(DE3) (Merck Millipore). Cells were grown at 37 °C to an optical density (600 nm) of ~0.8. Heterologous gene expression was switched on by the addition of 0.4 mM isopropyl β-D-1-thiogalactopyranoside and growth was then continued at 37 °C for a further 4 h before recovering the bacteria. The recombinant proteins were subsequently purified to near homogeneity using standard procedures. Specifically, the cell-free extract was subjected to immobilized metal affinity chromatography using a nickel column (Merck Millipore) in accordance with the manufacturer’s instructions. The level of endotoxin was reduced to <0.001 units/mL using EndotoxinOUT™ resin (G-Biosciences, St. Louis, MO, USA). Protein concentrations were estimated using Bradford reagent (Bio-Rad Laboratories Inc., Hercules, CA, USA).

### 4.9. Statistics

Data were analyzed by one-way ANOVA followed by Tukey–Kramer post hoc tests.

## 5. Conclusions

In conclusion, we demonstrated that Trp depletion enhances high-affinity Trp uptake not only into cells with added TrpRS protein but also into TrpRS-overexpressing cells. Moreover, we clarified that tryptophanyl-AMP production by TrpRS is crucial for high-affinity Trp uptake. Further studies are required to elucidate the molecular mechanism of high-affinity Trp uptake via enzymatic production of tryptophanyl-AMP by TrpRS. In addition, we found that Torin-1 treatment suppresses high-affinity Trp uptake. Therefore, it is also important to investigate the relationship between Trp starvation-specific phenomena, such as frame-shifting at Trp codons and Trp-to-Phe codon reassignment, and high-affinity Trp uptake.

## Data Availability

The data that support the findings of this study are available from the corresponding author upon reasonable request.
